# Development and Characterisation of a Whole Hybrid Sol-Gel Optofluidic Platform for Biosensing Applications

**DOI:** 10.3390/nano12234192

**Published:** 2022-11-25

**Authors:** Emma MacHugh, Graceson Antony, Arun Kumar Mallik, Alicja Kaworek, Declan McCormack, Brendan Duffy, Mohamed Oubaha

**Affiliations:** 1 School of Chemical and Pharmaceutical Sciences, Technological University Dublin, City Campus Grangegorman, D07 H6K8 Dublin, Ireland; 2 Centre for Research in Engineering Surface Technology (CREST), FOCAS Institute, Technological University Dublin, 13 Camden Row, D02 HW71 Dublin, Ireland; 3 School of Physics and Clinical and Optometric Sciences, Technological University Dublin, City Campus Grangegorman, D07 H6K8 Dublin, Ireland; 4 Centre for Industrial and Engineering Optics (IEO), FOCAS Institute, Technological University Dublin, Camden Row, D07 H6K8 Dublin, Ireland; 5 Photonics Research Centre, Technological University Dublin, City Campus Grangegorman, D07 H6K8 Dublin, Ireland

**Keywords:** organic-inorganic sol-gel materials, photolithographic fabrication, optical devices, microfluidic, waveguides, biosensing

## Abstract

This work outlines, for the first time, the fabrication of a whole hybrid sol-gel optofluidic platform by integrating a microfluidic biosensor platform with optical waveguides employing a standard photolithography process. To demonstrate the suitability of this new hybrid sol-gel optofluidic platform, optical and bio-sensing proof-of-concepts are proposed. A photoreactive hybrid sol-gel material composed of a photopolymerisable organically modified silicon alkoxide and a transition metal complex was prepared and used as the fabrication material for the entire optofluidic platform, including the optical waveguides, the sensing areas, and the microfluidic device. The most suitable sol-gel materials chosen for the fabrication of the cladding and core of the waveguides showed a RIC of 3.5 × 10^−3^ and gave thicknesses between 5.5 and 7 μm. The material was optimised to simultaneously meet the photoreactive properties required for the photolithography fabrication process and the optical properties needed for the effective optical operability of the microstructured waveguides at 532 and 633 nm with an integrated microfluidic device. The optical proof-of-concept was performed using a fluorescent dye (Atto 633) and recording its optical responses while irradiated with a suitable optical excitation. The biosensing capability of the platform was assessed using a polyclonal primary IgG mouse antibody and a fluorescent labelled secondary IgG anti-mouse antibody. A limit of detection (LOD) of 50 ug/mL was achieved. A correlation between the concentration of the dye and the emission fluorescence was evidenced, thus clearly demonstrating the feasibility of the proposed hybrid sol-gel optofluidic platform concept. The successful integration and operability of optical and microfluidic components in the same optofluidic platform is a novel concept, particularly where the sol-gel fabrication material is concerned.

## 1. Introduction

Currently, the majority of medical diagnostic testing takes place in centralised hospital laboratories, which is time intensive and utilises large-scale and expensive equipment. Over the past two decades, the significant increase in biosensors development has sought to address this situation by enabling point-of-care (POC) testing on inexpensive disposable biosensor chips. Many relevant studies and reviews have recently summarised the state-of-the art biosensor field [1,2,3], the most popular being so far electrochemical [4,5], photoelectrochemical [6,7], and optical [8,9]. However, the currently available POC devices are typically designed to diagnose just one diagnostic parameter. For example, diabetes care has been revolutionised by the introduction of glucose meters, but in other diagnostic situations single analyte testing is not sufficient. This is the case where the routine early detection of multiple diseases (cancers, cardiac enzymes) and monitoring of several analytes in parallel (e.g., glucose, cholesterol, triglycerides, etc.) is required. This deficiency has been clearly identified in a recently published report on the current trends of the biosensor market [10], which further validated the findings of a previous report written in 2007 by Frost and Sullivan [11], where a strong requirement for multianalyte biosensors, capable of delivering real-time diagnostic feedback on a minimum of eight analytes, was already underlined. This situation is largely due to the difficulty to integrate on a same platform driving materials, such as microfluidic devices, multiple sensing components, such as independent sensing windows, and high detection methodologies, such as optical devices. 

The approach proposed here consists of a demonstration of concept for the fabrication of an optofluidic biosensor system that would enable the simultaneous integration of the various components required for sensing application using the same material and fabrication process.

Optofluidics is the interdisciplinary research field that integrates both optical and microfluidic technologies to target performances and applications that the individual technologies cannot attain [12]. The combination of both an optical and a microfluidic component onto a single device, has enabled the exploitation of each field to open up a wide range of applications, the most popular reported so far are for photonics, sensing and imaging, healthcare, food and energy [13,14,15,16,17]. The integration of microfluidics into photonic devices provides a way to tune and reconfigure microphotonic devices at the micrometre scale, a property they lack on their own. Microfluidics also offer a wealth of different ways to control microphotonic devices, laminar flows can transport various nanostructures or species that have desired optical properties efficiently [18]. Optofluidic devices are well suited, in particular, for biological or chemical analysis and detection in extremely small volumes because of the integration of sample preparation and delivery with the analytical mechanism [19,20,21,22]. However, to date, many of the fabrication process of these systems utilise polydimethylsiloxane (PDMS) as a common fabrication material, generally for the fabrication of the microfluidic components [17,18,23,24,25], although systems fabricated completely using PDMS have been reported [26]. Drawbacks to using PDMS include swelling of the material due to the absorption of organic solvents or hydrophobic molecules [27]. Deformation of microchannels can occur due to solvent induced swelling [28]. The inherent hydrophobic nature of PDMS can also pose problems for the control of fluids, in particular towards biosensing and biomedical applications [29,30]. Various contact angle measurements have been reported for untreated PDMS ranging from 105° to 113.5° [31,32]. PDMS for the fabrication of microfluidic or optical components also requires the use of a master mould to create the structures which limit resolutions of the channels and can be time consuming [26]. In this context, the development of innovative materials and fabrication methodologies that would facilitate the integration of the fluidic and optical systems are needed.

Over the past two decades, the sol-gel process has been one of the popular routes for the development of multifunctional nanomaterials due to its ability to combine both organic and inorganic compounds in a liquid phase, at low temperatures and using eco-friendly precursors [33]. This process enables the achievement of homogeneous, and morphology structured nanosize objects with tuneable properties, including optical, mechanical, thermal, and others. Due to the hybrid character of these family of materials and their inherent liquid nature, they can be processed as thin films and further photostructured as microstructures, of great interest for the fabrication of miniature multifunctional devices. One of the recently reported concepts consists of exploiting photoreactive hybrid sol-gel materials for the parallel and direct fabrication of both systems onto the same substrate employing a standard photolithography process [29]. Hybrid sol-gel materials are attractive materials to use as dual fabrication materials owing to their increased chemical and mechanical properties which can be attributed to the inorganic–organic hybrid nature of the material [34]. They also possess tunable optical properties, such as refractive index and absorption [35,36]. These materials have shown promise towards a variety of sensing applications, including optical sensors for O_2_ [37,38] and water vapour sensing [39], and in photonic sensors for the detection of biofilm formation [40]. As negative photoresists they eliminate the need for a master mould to be used and, therefore, are suitable for low cost production of optofluidic or microfluidic devices [29].

Here, the optofluidic platform is based on the integration of two systems: a microfluidic biosensor platform and planar optical waveguides fabricated onto the same substrate. The fabrication material for both systems is fabricated via a standard photolithography process, employing a stable photoreactive silicate/zirconium hybrid sol-gel material. The optical sensing capabilities of the platform are tested using a fluorescent dye, Atto 633. The capabilities of the platform towards bio-sensing will be carried out using a polyclonal primary IgG mouse antibody and a fluorescent labelled secondary IgG mouse antibody. Correlation between the optical performances of the platform and the concentrations of the dye will prove the demonstration of the proposed concept. 

## 2. Materials and Methods

### 2.1. Design of the Optofluidic Platform 

This work proposes the development of an innovative optofluidic device that will also take advantage of the versatility of the sol-gel technology in tuning the functionality and optical properties of the materials. 

**Figure 1:**
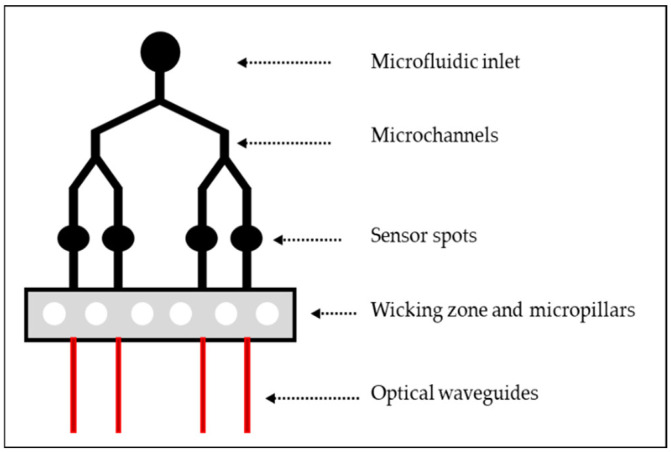
Schematic representation of the optofluidic platform.

In researching monolithic hybrid sol-gel optofluidic devices, our investigations have led us to the design of an innovative waveguide-based optofluidic device that will integrate optical waveguides onto a microfluidic platform and sensor spots, as sketched in Figure 1. The microfluidic platform is comprised of an inlet, y split microfluidic channels (MFCs), 4 sensor spots and a wicking zone which contains micropillars. The wicking zone will act as a waste reservoir for any excess fluid and the micropillars will aid in drawing the liquid through the MFCs and help support the PDMS cap from collapsing into the wicking zone. The platform would preferably be designed as a single-use device with the wicking zone acting as a waste reservoir that would negate the need for an outlet port on the platform. As the platform was fabricated from two separate photomasks, the optical waveguides were fabricated to lie within the wicking zone rather than the sensing spots of the microfluidic platform. Ideally, the platform would be fabricated from a single photomask consisting of the whole optofluidic platform, with the waveguides situated within the sensing spots of the microfluidic section of the photomask. 

Unlike microfluidic devices, where the only required property for the material is a high surface energy, light propagation within optical waveguides can only occur when total internal reflection (TIR) is taking place at the interface between the core and the cladding of the waveguide. This phenomenon depends on the optical properties of the materials, primarily the refractive index contrast (Δn) between the core of the waveguide and its cladding. Wavelength can impact TIR due to index dispersion [41,42,43,44]. TIR and self-consistency of the wave in the guide are the two conditions for light transverse confinement in the core region. The confined light can propagate along the waveguide axis and is then called a propagation mode. The higher the index difference, the core transverse size, and the optical frequency, the higher the confinement and thus the number of propagation modes. The 2D intensity profile and the associated effective index of each mode can be computed for a 3D waveguide by solving Maxwell’s or Helmoltz’s equations using numerical schemes as finite differences or finite elements methods. According to results reported in these studies waveguiding in the visible domain is possible when Δn comprises between 3 × 10^−3^ and 10 × 10^−3^ for tetragonal micron scale waveguides [41,42,43,44]. The nature of the propagating modes (single or multimode) would be further defined by the size and shape (rectangular or squared) of the tetragonal waveguides, but in all cases the light propagation within the core of the waveguide would take place.

### 2.2. Materials Synthesis

These materials are prepared employing a three-step sol-gel synthesis of two photoreactive alkoxide precursors, an organically modified silicon (MAPTMS) and a zirconium complex from the chelation of zirconium propoxide by methacrylic acid, as reported elsewhere by the authors [45]. In order to make the sol-gel photocurable, a photo-initiator—Irgacure 184 was used and fixed at a concentration of 5 mol. % against MAPTMS and the total theoretical hydrolysis degree for all formulations fixed at 50%. 

It is important to note that precise control of the optical properties of the materials is essential for the light propagation within the optical waveguide. Indeed, optical simulations performed in a related study have shown that the propagation of the light in optical waveguides relies on both the size, operating wavelength, and refractive index contrast (RIC) between the core and the cladding of the waveguide [40]. For waveguides having a cross-section in the range 4 to 6 microns and operating between 532 and 633 nm, the minimum RIC would be 0.003. In order to achieve this RIC, a set of four different hybrid sol-gel materials were prepared by altering the relative concentration of the two precursors, as shown in Table 1.

### 2.3. Fabrication Process

**Figure 2:**
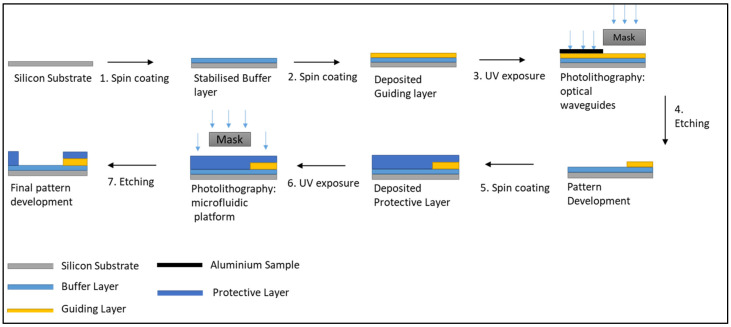
Schematic of the fabrication process for the optofluidic platform.

The fabrication process of the optofluidic platform is sketched in Figure 2. It involves the consecutive microstructuration of the optical waveguides (step 1 to 4) and the microfluidic counterpart (steps 5 to 7) on the same substrate.

The optical waveguides are prepared as follows: (1) the cladding layer was spin-coated at 900 rpm onto a silica wafer and subsequently fully stabilised by UV-irradiation for 500 s. (2) The core layer was deposited using the same spin-speed and partially thermally stabilised for 10 min at 100 °C in an oven to make it touch dry while maintaining its photoreactive properties. (3) The optical waveguides were photo-patterned employing a photomask. Prior to UV-irradiation, a portion was “blocked off” using a sample of aluminium so that the MFCs could be subsequently patterned in this unexposed area. (4) Following the irradiation step, the sample was immersed in isopropanol for 1 min to enable etching of the non-exposed areas of the coating, thus revealing the microstructured patterns. (5) A second layer of the cladding layer was processed with the same conditions as the core layer. Importantly, in addition to acting as a cladding layer to enable the symmetrical RIC around the core of the waveguide, this cladding layer will be employed for the microstructuration of the microfluidic protective layer. At this stage of the process, the microfluidic photomask was employed. Care was taken to ensure that the output of the optical waveguides was positioned at the entry of the wicking zone. (6) The sample was then photo-patterned using the mask aligner for 500 s. (7) The sample was then sonicated in IPA for 1 min and rinsed with IPA to remove any residual sol-gel debris issued from the etching process and finally placed in an oven at 100 °C for 2 min to evaporate any remaining solvent.

### 2.4. Techniques of Characterisation

#### 2.4.1. Refractive Index Measurements

The refractive index (RI) for each material was measured by prism-coupling using the Metricon 2010 (Metricon Corporation, Pennington, NJ, USA). Prior to RI measurements, the sol-gel materials were spin coated onto a silicon wafer at a spin speed of 900 rpm to achieve coating thicknesses close to 5 microns. The samples were subsequently cured at varying UV irradiation times from 0 to 650 s employing a UV-irradiation system (Kloé UV KUB2) (Kloe, Montpellier, France) The coatings were then dried at 100 °C for 10 min to ensure the coatings were fully touch dry. 

#### 2.4.2. SEM and Optical Microscopy

Scanning electron microscopy (SEM) images were recorded employing a Hitachi SU-70 SEM (Hitachi, Tokyo, Japan) using an accelerating voltage of 5 keV for SEM images. Samples coated onto Si wafers were cleaved and sputter coated with 6.1 nm of platinum/palladium coating using a Cressington 208HR sputter coater (Cressington, Whatman, UK) to reduce the charge of the samples. Optical microscopy images of the sensor platform were imaged using a Keyence VHX digital microscope (Keyence, Milton Keynes, UK).

#### 2.4.3. UV-Vis Spectroscopy

A UV-Vis absorption spectrum for an aqueous solution of Atto 633 was recorded on a Perkin Elmer Lambda 800 spectrometer (Perkin Elmer, Waltham, MA, USA). Fluorescence spectroscopy was also recorded for the dye and the emission spectrum was recorded using a Perkin Elmer LS 55 Luminescence Spectrometer (Perkin Elmer, Waltham, MA, USA). The excitation wavelength chosen was specific to the dye. Emission spectra were recorded between 650 and 900 nm with an excitation wavelength of 633 nm.

### 2.5. Optical Sensing Characterisation

**Figure 3:**
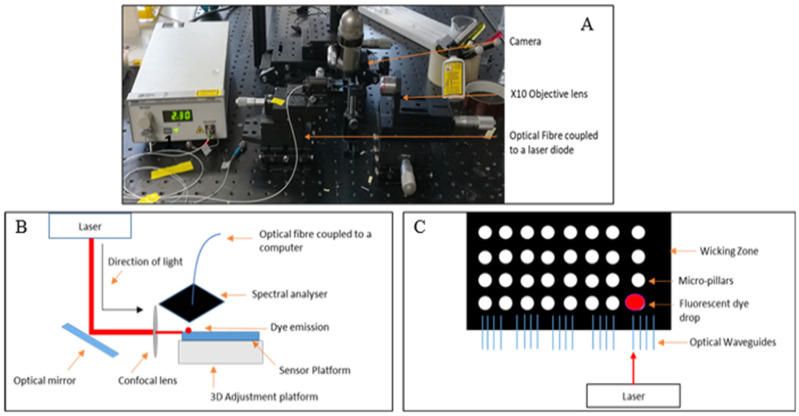
Optical set up for waveguide testing (**A**), schematic of the optical set up used for the fluorescence studies (**B**), and schematic of the sensor platform with the location of the fluorescent dye (**C**).

The operability of the optical waveguides was initially tested by coupling the optical waveguides to a single mode laser diode tuned at 635 nm at an intensity of 2.5 mW, through an optical fibre (Figure 3A). To ensure the output from the waveguides was visible it was magnified using a ×10 objective lens. 

Having successfully observed that the light was propagating within the core of the waveguides, with an optical loss of 0.1 dB/cm (measured by cut-back), the following step consisted of evaluating the effectiveness of the optical excitation within the wicking zone by employing well-known fluorescent dye, Atto 633 in various concentrations.

Atto 633 was deposited in the wicking zone of the platform and subsequently irradiated with a red laser line at 632.8 nm (Uniphase He/Ne laser) with an intensity of 0.1 mW. A schematic of the optical set up used is sketched in Figure 3B. The emission spectra were collected via a spectral analyser (Avantes AvaSpec-2048) (Avantes B.V, Apeldoorn, The Netherlands) with a broad band light source (AvaLight HAL-S) (Avantes B.V, Apeldoorn, The Netherlands) guided by a fibre optic cable (Avantes FC-UV400-2) (Avantes B.V, Apeldoorn, The Netherlands). A schematic of the sensor platform with the location of the fluorescent dye is sketched in Figure 3C. As the optical fluorescence set up was adapted to be used with optical waveguides, notch filter passes were not used for fluorescence detection. 

### 2.6. Biosensing Protocol 

In order to assess the suitability of the optofluidic platform as a biosensor, an immuno-fluorescence technique was employed, and a primary antibody-mouse IgG and a fluorescent labelled secondary antibody anti-mouse IgG labelled with CF 555 (Merck Life Science UK Limited, Gillingham, UK) were used. 

**Figure 4:**
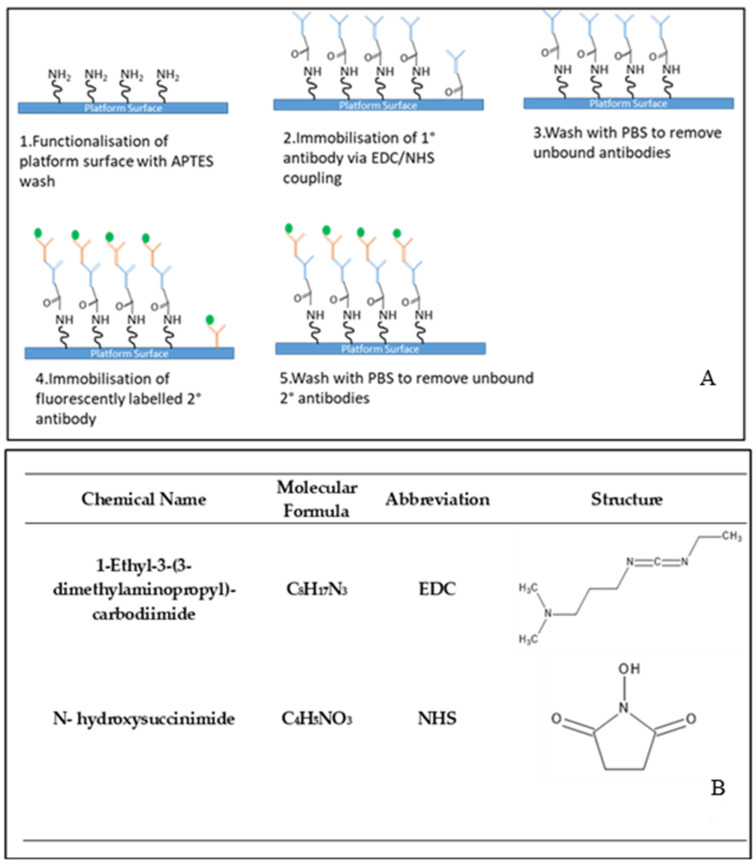
Representation of the biotechnological protocol employed in the sensing testing of the optofluidic device (**A**) and chemical name, molecular formula, common abbreviation and structure of the coupling agents used (**B**).

The bio-technology protocol was established to demonstrate the applicability of the developed optofluidic device as a sensing platform (Figure 4A). 1-Ethyl-3-(3-dimethylaminopropyl)-carbodiimide (EDC)/N-hydroxysuccinimide (NHS) coupling chemistry is used to bind the primary antibody to the functionalised surface of the sensor platform and is a widely used immobilisation strategy [46]. EDC is a water-soluble cross-linking agent which forms amide bonds between carbonyl and amine groups. As EDC is not fully stable in water, due to the oxygen atoms acting as nucleophiles and inactivating the EDC, NHS is used to improve its stability [47]. The structures of both compounds are shown in Figure 4B. 

The platform was first functionalised using a dilute APTES wash at 5% concentration. In order to ensure the primary antibody was bound to the surface of the sol, EDC/NHS coupling chemistry was carried out. The IgG was reconstituted into a 1 mg/mL concentration, by dissolving 5 mg of IgG in 5 mL of phosphate-buffered solution (PBS). 

The EDC/NHS mixture was prepared as follows: 1.12 mg of EDC was dissolved in 1 mL of PBS, and 1.06 mg of NHS was dissolved in 1 mL of PBS to ensure both compounds were in excess in relation to the antibody concentration. The EDC was then added to the antibody followed by the NHS. Then, 5 µL of this mixture was then pipetted onto the sensor spots of the optofluidic platform. The IgG primary antibody was left to bind to the surface by diffusion for 2 h before being washed with PBS 3 times to remove any excess unbound primary antibody. 

Fluorescent labelled secondary antibody solutions were prepared at concentrations of 10, 50, 100, 200, and 500 µg/mL. From each solution, 5 µL were pipetted onto the corresponding functionalised spot with the primary antibody and left to bind for 2 h. Following this binding step, the surface was rinsed 3 times with PBS to eliminate any non-surface bound antibody. A blank was also prepared on the same platform to identify any non-specific binding that may occur of the primary antibody to the platform itself, without using EDC/NHS coupling chemistry. The highest concentration of the secondary antibody was pipetted onto the corresponding area that had not been functionalised via the EDC/NHS coupling chemistry. 

## 3. Results and Discussion

### 3.1. Refractive Index Measurements

The four-hybrid sol-gel materials A to D were processed as coatings at a spin-speed of 750 rpm and stabilised by UV irradiation at various times (0, 50, 100, 250, 500, and 650 s). The objective of this study was to identify the optimum curing time required to fully stabilise the structure of the materials and, therefore, achieve stable optical properties and coating thicknesses. Having obtained this information, it would be possible to determine the materials to be used as the core and cladding of the optical waveguides that satisfy the RIC of 0.003, as discussed above. 

**Figure 5:**
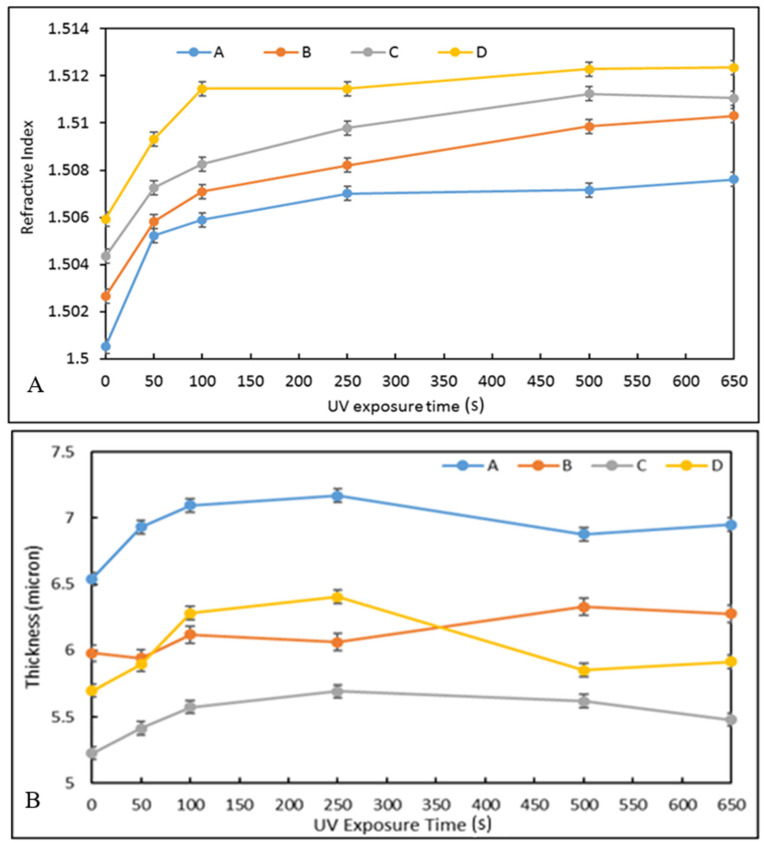
Refractive index vs. UV curing time for materials A–D (**A**) and coating thickness vs. UV exposure time for all materials A–D (**B**).

The RI values for each formulation is plotted against the UV irradiation time (Figure 5A). For all materials, it can be observed that the RI increases rapidly from 0 to 250 s UV-irradiation and then stabilises within the measurement errors for higher exposure times, achieving RI values of 1.507, 1.509, 1.5097, and 1.512 for materials A–D, respectively. It is noteworthy that as the concentration of zirconium complex increases, the value of the RI increases regardless of the UV-exposure time. Additionally, the variation of the RI from the lowest to the highest exposure times decreases regularly as the concentration of the zirconium complex is increased, 7.5 × 10^−3^, 7.1 × 10^−3^, 6.3 × 10^−3^, and 6.2 × 10^−3^, for materials A to D, respectively. This suggests that the zirconium complex is the main element governing the RI value and that it limits the photopolymerisation process, probably by acting as a network hardener to the silicate matrix. 

It can be noted that the most suitable pair of materials for the fabrication of the cladding and core of the waveguides would be materials B and D, respectively, as they show an RIC of 3.5 × 10^−3^.

The thickness of the coatings was also measured as a function of UV curing time (Figure 5B). The thickness of the coatings was measured using a Metricon 2010 prism coupler. It can be observed that all coatings swell with an average value of 5% against their initial thickness, achieving thicknesses comprised between 5.5 and 7 μm. Importantly, the full stabilisation of the coating thicknesses is achieved for UV-irradiation times of 500 s. This demonstrates that the physico-chemical phenomena involved in UV-exposure are fully completed after 500 s of exposure and this duration is necessary for the fabrication process of the optofluidic platform.

Owing to these results and to identify the suitable deposition speeds to achieve the required coating thicknesses, as identified in the design of the platform (see Section 2.1), both BL and GL materials were deposited at deposition speeds in the range 500–2000 rpm and exposed to UV-irradiation for 10 min to enable fabrication of touch-dry and fully stable coatings. The coating thicknesses were then measured by the prism-coupling technique and obtained results are presented in Table 2.

The difference of the coating thickness could be explained by the differences in the viscosities of both sols. Indeed, the results suggest that the increase in zirconium complex would tend to increase the fluidity of the sol, thus decreasing the thickness of the coatings deposited. The difference in the evolution of the coating thickness as function of UV exposure time is intimately linked to the double capability of polymerisation of the developed materials; radical organic polymerisation (via unsaturated methacryloxy groups) and inorganic polycondensation (via the silicon and zirconium alkoxide groups). Until 250 s of UV exposure, the organic polymerization process is favoured resulting in the swelling of the coating. Here, the energy provided by the UV exposure is directly used by UV sensitive moieties resulting in an overall swelling of the coatings. When the organic polymerisation is complete, the inorganic polycondensation process starts to take place by exploiting the heat energy provided by the UV exposure and not being used by the organic moieties. Unlike organic polymerisation, inorganic polycondensation processes tend to decrease porosity and increase condensation of the materials, resulting in the decrease in coating thicknesses. This process is favoured by the presence of zirconium, probably due to its higher condensation capability, explaining why the thickness decrease is more pronounced as the zirconium concentration increases.

### 3.2. SEM Analysis

**Figure 6:**
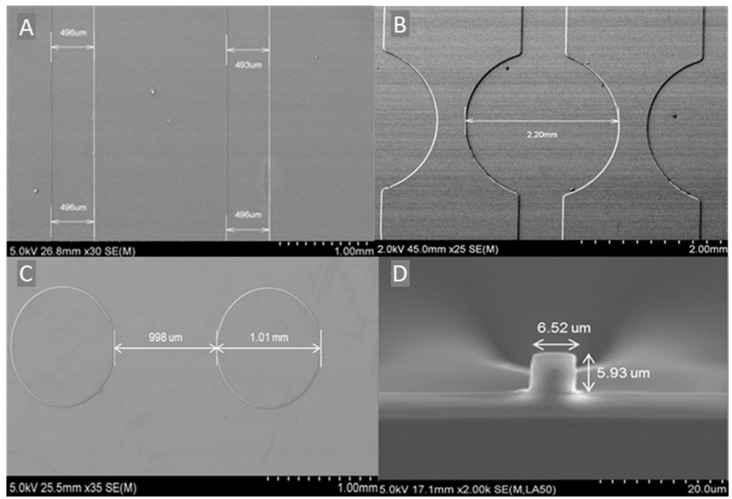
SEM images of (**A**) MFCs, (**B**) sensor spots, (**C**) pillars, and (**D**) cross-section of the optical waveguides.

Figure 6A shows the SEM image of the MFCs with a width of 495 μm (±2 μm). The 5 μm difference to the employed photomask (500 μm) is attributed to the measurement error of the SEM, due to the difficulty to focus precisely on the edge of the MFCs. Nevertheless, this difference corresponds only to 1%, which confirms a good control of the established photolithography process. This is further confirmed by the linear shape of the MFCs. Figure 6B represents the SEM image of the sensor spots and their junction with the MFCs. Their good circular shape, with a diameter of 2.2 mm (matching the photomask size with a resolution of 10%) and their direct integration to the MFCs indicates that the fabrication process is adequate for the development not only of linear microstructures but also for microstructures of complex shapes. The pillars located in the wicking zone are shown in Figure 6C and are found to exhibit diameter of 1 mm and separated between each other by 1 mm, as expected from the photomask. Figure 6D shows the SEM images of the cross-section of the optical waveguide along with the buffer layer on the silicon wafer. From the cross-section view, it can be observed that the optical waveguides exhibit a quasi-squared profile with a section of 6 μm (±10%) and a buffer layer close to 4.5 μm.

Owing to these results, it can be concluded that the developed photolithography fabrication process is suitable for the fabrication of residue-free microstructures with the expected dimensions.

### 3.3. Optical Waveguide Testing

**Figure 7:**
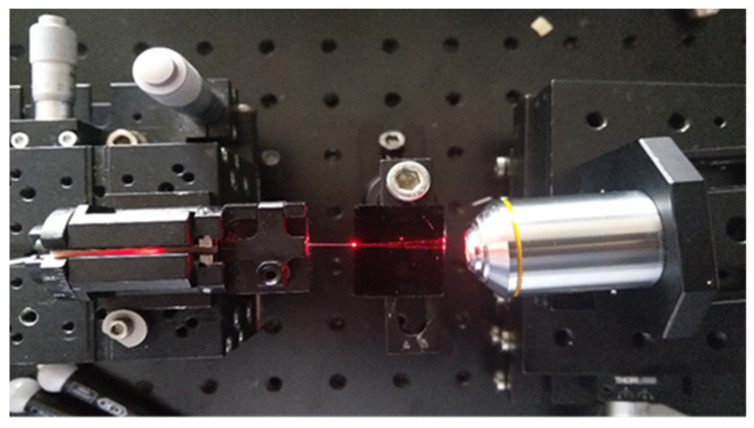
Top view of the light propagation within the waveguide.

The waveguides were tested to ensure the light was properly propagating within the waveguides by coupling a 635 nm laser diode to the waveguides input and characterising both surface and output (Figure 7). The length of propagation was 50 mm. Although no measurement of the optical performances of the waveguides were performed (optical losses and modality), the light is clearly propagating in the core of the optical waveguides, suggesting that the waveguide may be operating in a single-mode manner. Therefore, this result demonstrates that the defined RIC is effectively sufficient to avoid important light dissipation within the cladding. This ensures a suitable excitation of the fluorescent dyes and fluorescently labelled antibodies that are expected to be located at the output of the waveguides for the sensing and biosensing testing of the optofluidic platform.

### 3.4. UV-Visible Absorption and Fluorescence Spectroscopy

Prior to testing the Atto 633 dye on the optofluidic platform, it was necessary to define its absorption and fluorescence spectra, including the optimum excitation wavelength (λ_max_).

**Figure 8:**
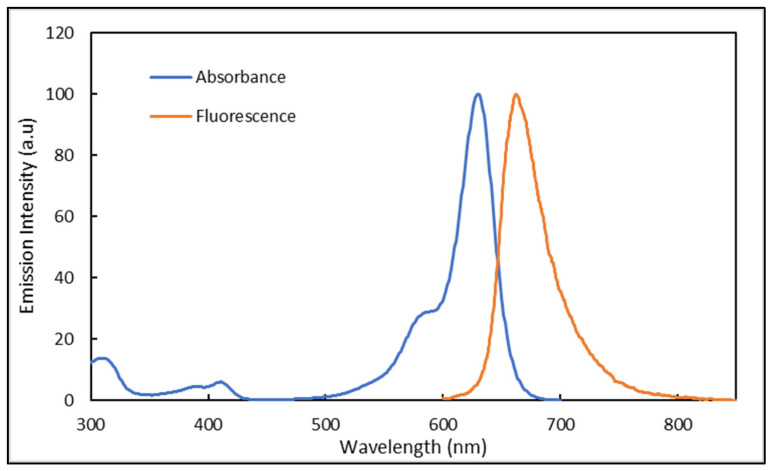
Absorbance and emission spectra of Atto 633 dissolved in water and a concentration of 1.2 × 10^−5^ g/mL. Emission recorded with an excitation at 633 nm.

At a concentration of 1.2 × 10^−5^ g/mL, the Atto 633 dye exhibits a large absorption in the range 290–685 nm with two absorption bands located at 580 and 633 nm (λ_max_), as shown in Figure 8. The absorption below 500 nm accounts for less than 5% of the overall absorption. Employing an excitation wavelength of 633 nm, Atto 633 is found to emit in the red region from 650 to 820 nm, and shows a λ_max_ at 663 nm. The overlap between the emission and absorption comprises the spectral range between 600 and 680 nm. Therefore, any emission recorded outside this spectral range can only be attributed to fluorescence phenomena with no effect of the absorption.

### 3.5. Optofluidic Platform Testing

The fabricated optofluidic platform was tested using the Atto 633 dye at two different concentrations (1.2 × 10^−5^ and 1.2 × 10^−6^ g/mL) excited with a laser tuned at 632 nm and a power of 0.1 mW. The purpose of testing two different concentrations was to identify the possible occurrence of non-radiative emission originating from competitive dye absorption.

**Figure 9:**
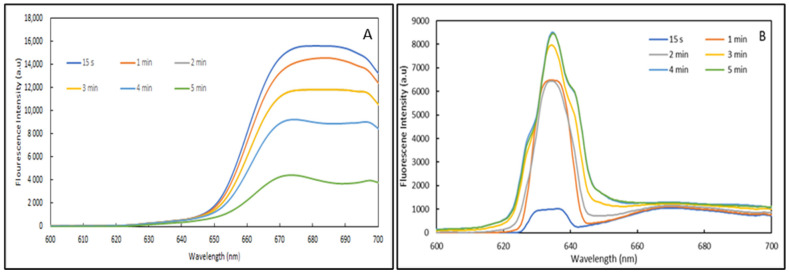
Emission spectra of Atto 633 dye at 0.1 mW at (**A**) 1.2 × 10^−5^ g/mL and (**B**) 1.2 × 10^−6^ g/mL.

The emission spectra for both concentrations are presented in Figure 9. At the highest concentration, the Atto 633 dye shows a broad emission band centred at 663 nm, which decreases in intensity over time. Interestingly, a weak emission is observed at 633 nm with a similar intensity for all exposure times due to the excitation source and suggesting the majority of the light is absorbed by the dye.

For the lower concentration, the emission spectra are clearly found to rely on the exposure time. Indeed, although all spectra show two emission bands located at 633 and close to 670 nm along with a small shouldering around 600 nm, samples exposed for durations comprised between 3 and 5 min show an additional two bands located at 627 and 640 nm. Importantly, the emission band located at 633 nm and the shouldering around 600 nm are found to increase with exposure time.

Owing to these results, one can assume that the main difference in terms of emission behaviour for the two concentrations can only relate to optical phenomena that are sensitive to the proximity of the dye molecules in the sol-gel matrix. Indeed, for the high dye concentration, the proximity of the dye within the sol-gel matrix is reduced, thus providing opportunities for energy transfer between the molecules, a phenomenon known as non-radiative emission (NRE) [48]. It may be possible that because of the continuous radiation the NRE phenomenon increases and provokes an overall decrease in the detected fluorescence, as observed for this sample. For the lower dye concentration sample, as the dye molecules would be further from each other, the NRE phenomenon may be reduced. Conversely, as emissions are observed at higher energies it is likely that an optical phenomenon, called excited states absorption (ESA), is taking place [49]. More precisely, ESA phenomena consists of energy transfer with the same or different energy to an already excited level that will reach an even higher energy level. In the present case, it is likely that energy levels observed at 633 nm may receive energy from energy levels at 670 nm to produce an emission centred at 633 nm. This hypothesis is reinforced by the fact that the emission around 670 nm also increases within the exposure time.

Although, the emission properties of Atto 633 dye differ when used on our platform, it is still possible to record an emission close to 670 nm, characteristic of this dye.

In summary, the results show that the developed optofluidic device has the potential to act as a waveguide-based fluorescent biosensor platform. The next section will investigate the potential of our platform towards bio-sensing applications, where the detection of fluorescent labelled antibodies usually employed in medical diagnostic applications will be performed.

### 3.6. Biosensing Testing

An IgG mouse primary antibody was immobilised onto the surface of the sensor spots. Firstly, the surface was functionalised using a dilute 5% APTES wash, ensuring a hydrophilic surface (contact angle of 44°). The contact angle of the naive material (BL) was measured at 59°. The primary antibody was bound using an EDC/NHS coupling agent. The fluorescent-labelled secondary antibody (CF 555) was then pipetted onto the previously bound primary antibodies and left to bind via diffusion for 1 h. The platform was then washed with PBS to ensure residual unbound antibodies were removed, thus minimising undesired emissions. To investigate the concentration effect and eventually obtain a correlation between the concentration and the emission of fluorescently labelled antibodies, five concentrations were prepared, 500, 200, 100, 50, and 10 µg/mL, and each concentration of antibodies was bound to a different sensor spot on the platform.

**Figure 10:**
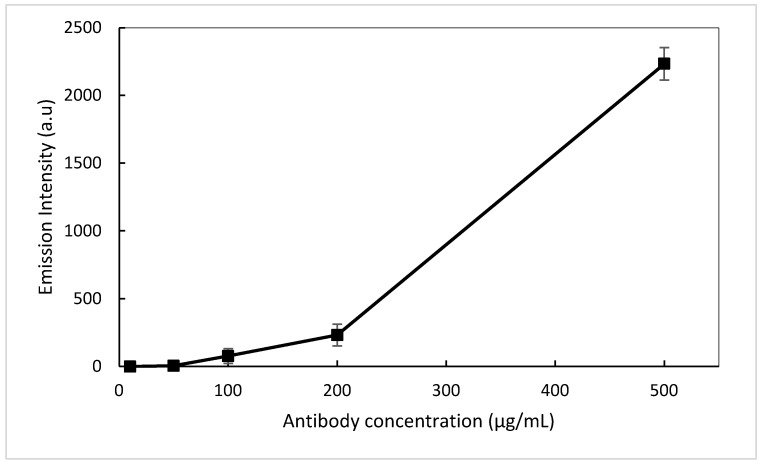
Antibody concentration curve @ 574 nm.

As CF 555 shows a broad absorption in the 550–565 nm spectral range (not shown here), a green laser tuned at 532 nm was selected as the excitation source and was applied to each individual sensor spot with a pulse rate of 3 s per spot to minimise any photobleaching of the fluorescent labelled antibody. The change in fluorescence was captured via a spectrometer and the subsequent concentration curve plotted in Figure 10. As the antibody concentration increases, a progressive increase in the fluorescence intensity is captured. A limit of detection (LOD) of 50 µL/mg was achieved. Although the LOD is high compared to the current state-of-the-art biosensor devices, these results enabled us to demonstrate the concept of a waveguide-based hybrid sol-gel biosensor as outlined in this paper. Future work would focus on testing intermediate antibody concentrations between 200–500 ug/mL and above 500 ug/mL to further improve the LOD.

## 4. Conclusions

This work focused on the fabrication and proof-of-concept of a novel waveguide-based optofluidic device employing photoreactive hybrid sol-gel materials and a standard photolithography process. The optofluidic platform was fabricated using two different sol-gel formulations developed to meet the optical properties of waveguides operating in the visible domain, which can be used for exciting fluorescent dyes. The capability of the platform as an optical sensor was tested using a red laser line to excite various concentrations of a fluorescent dye (Atto 633). The capability of the platform towards optical bio-sensing was also investigated using a polyclonal primary IgG mouse antibody and a fluorescent labelled secondary IgG mouse antibody and the results achieved demonstrate that the newly developed platform has the ability to act as a biosensor device. The main advantage of this novel platform lies in its versatility, as the sol-gel materials proposed may be modified depending on the specific analyte to be detected. Although the proof-of-concept of waveguide-based hybrid sol-gel optofluidic biosensors was demonstrated for the first time, further investigations are required to advance the technology towards high performing biosensor systems, including optimisation of the optical setups, optical characterisations, including permittivity and propagation properties, as well as biotechnological protocols. The technology would also strongly benefit from the development of advanced photoreactive super-hydrophilic materials for the rapid flow of analytes within the microfluidic system and optical coupling methodologies for efficient light laser/optical waveguides coupling, which constitute, today, probably the major limitations of this platform.

## Figures and Tables

**Table 1:** Sol-gel formulations for the materials used in the fabrication of optical waveguides.

Material Name	MAPTMS (mol. %)	ZPO/MAAH (mol. %)
A	84.39	15.61
B	82.65	17.35
C	81.64	18.36
D	80	20

**Table 2:** Thickness versus deposition speed for materials BL and GL.

Spin Speed (rpm)	Thickness BL (μm)	Thickness GL (μm)
500	9.5 ± 0.50	9.0 ± 0.40
750	6.0 ± 0.45	5.7 ± 0.35
900	4.5 ± 0.45	4.1 ± 0.30
1000	3.6 ± 0.30	3.3 ± 0.30
1100	3.0 ± 0.25	2.5 ± 0.25
1500	2.1 ± 0.20	1.6 ± 0.20
2000	1.6 ± 0.10	1.2 ± 0.1

## Data Availability

Not applicable.
